# Elucidating Regulatory Mechanisms of Genes Involved in Pathobiology of Sjögren’s Disease: Immunostimulation Using a Cell Culture Model

**DOI:** 10.3390/ijms26125881

**Published:** 2025-06-19

**Authors:** Daniel D. Kepple, Thomas E. Thornburg, Micaela F. Beckman, Farah Bahrani Mougeot, Jean-Luc C. Mougeot

**Affiliations:** 1 Translational Research Laboratories, Department of Oral Medicine/Oral & Maxillofacial Surgery, Atrium Health Carolinas Medical Center, Charlotte, NC 28203, USA; daniel.kepple@atriumhealth.org (D.D.K.); thomas.thornburg@atriumhealth.org (T.E.T.); micaela.beckman@atriumhealth.org (M.F.B.); 2 Department of Otolaryngology/Head & Neck Surgery, Wake Forest University School of Medicine, Winston-Salem, NC 27101, USA

**Keywords:** Sjögren’s disease, iSGEC, gamma-IFN, poly(I:C), ETS1, STAT1, Interleukin-33, miRNA, regulation

## Abstract

Sjögren’s disease (SjD) is an autoimmune disease of exocrine tissues. Prior research has shown that ETS proto-oncogene 1 (ETS1), STAT1, and IL33 may contribute to the disease’s pathology. However, the regulatory mechanisms of these genes remain poorly characterized. Our objective was to explore the mechanisms of SjD pathology and to identify dysfunctional regulators of these genes by immunostimulation of SjD and *sicca* relevant cell lines. We used immortalized salivary gland epithelial cell lines (iSGECs) from Sjögren’s disease (pSS1) and *sicca* (nSS2) patients, previously developed in our lab, and control cell line A253 to dose with immunostimulants IFN-γ or poly(I:C) (0 to 1000 ng/mL and 0 to 1000 µg/mL, respectively) over a 72 h time course. Gene expression was determined using qRT-PCR delta-delta-CT method based on glyceraldehyde-3-phosphate dehydrogenase (GAPDH) for mRNA and U6 small nuclear RNA 1 (U6) for miRNA, using normalized relative fold changes 48 h post-immunostimulation. Protein expression was quantified 72 h post-stimulation by Western blotting. Reference-based RNA-seq of immunostimulated pSS1 and nSS2 cells was performed to characterize the reactome of genes conserved across all used doses. The expression of ETS1 and STAT1 protein was upregulated (*p* < 0.05) in IFN-γ-treated pSS1 and nSS2, as compared to A253 cells. IFN-γ-treated nSS2 cell showed significant IL33 upregulation. Also, IL33 had a correlated (*p* < 0.01) U-shaped response for low-mid-range doses for IFN-γ- and poly(I:C)-treated pSS1 cells. RNA-seq showed 175 conserved differentially expressed (DE) genes between nSS2 and pSS1 immunostimulated cells. Of these, 44 were shown to interact and 39 were more abundant (*p* < 0.05) in pSS1 cells. Western blotting demonstrated nSS2 cells expressing ETS1 uniformly across treatments compared to pSS1 cells, despite similar mRNA abundance. miR-145b and miR-193b were significantly under-expressed in IFN-γ-treated nSS2 cells compared to pSS1 cells (*p* < 0.01). ETS1 and IL33 showed disproportionate mRNA and protein abundances between immunostimulated Sjögren’s disease-derived (pSS1), and sicca-derived (nSS2) cell lines. Such differences could be explained by higher levels of miR-145b and miR-193b present in pSS1 cells. Also, RNA-seq results suggested an increased sensitivity of pSS1 cells to immunostimulation. These results reflect current pathobiology aspects, confirming the relevance of immortalized salivary gland epithelial cell lines.

## 1. Introduction

Sjögren’s disease (SjD) is an autoimmune disease affecting exocrine tissues that affects up to three million people in the US [1] causing symptoms of dry mouth and eyes that can be detrimental to the patient’s quality of life [2]. SjD pathology can also lead to systemic complications including nephritis, peripheral neuropathy, and skin vasculitis [3]. SjD primarily affects women with an over 9:1 female/male ratio, with onset usually occurring between 45 and 55 years of age [4]. Factors that can affect onset include genetic predisposition, hormonal changes, abnormal innate and adaptive immune responses [5], viruses such as cytomegalovirus, Epstein–Barr virus, hepatitis C virus, human T-cell lymphotropic virus-1 and bacteria such as *Helicobacter pylori* [6,7,8]. These factors can stimulate Sjögren’s syndrome-related antigen A (SSA *alias* Ro60 RNA-binding protein) production. Currently there are no preventative or effective treatment options for SjD, with most treatments focusing on symptom management such as artificial saliva and eye drops rather than targeting pathological process. Several clinical trials attempted B-cell depletion and T-cell co-stimulation therapeutic approaches with no significant success [9,10,11].

Although the early stages of SjD remain poorly characterized, SjD pathogenesis involves damage to acinar cells from low levels of apoptosis caused by the loss of matrix metalloproteinases homeostasis within the salivary glands [12]. Further, extracellular matrix (ECM) damage from type I interferon (IFN) production by plasmacytoid dendritic cells (pDCs) promotes T-cell mediated hyperactivation of B-cell infiltrates in the ductal locations of the salivary gland [13,14]. Lymphoepithelial lesions may occur with or without the generation of detectable anti-nuclear autoantibodies against the 52 kDa and 60 kDa forms of anti-SSA in serum [15]. Therefore, the clinical diagnosis of anti-SSA-negative SjD is defined as having at least 50 inflammatory cell infiltrates in a 4 mm^2^ glandular section (one focus) in the salivary gland [16]. SjD patients negative for anti-SSA antibodies in serum may differ from anti-SSA-positive patients for some clinical features, notably having a lower risk for lymphoma development [17]. Patients with *sicca*, characterized by dry eyes and mouth [18], are at an increased risk of developing SjD, with approximately 10% of cases progressing to SjD [19]. The disease pathology in both *sicca* and SjD patients may occur from immune cell infiltrates accumulating in salivary glands influenced by the increased expression or activity of enzymes breaking down ECM collagen, namely matrix metalloproteinases MMP9 and MMP3 [9], causing sialadenitis, defined as salivary gland swelling, associated with acinar cell apoptosis and acini atrophy [20]. The activity of these MMPs is increased when the expression ratio of MMP9 and the tissue inhibitor of metalloproteinase 1 (TIMP1) in salivary gland epithelial cells (SGECs) of *sicca* and SjD patients is increased [15,21]. MMP9 glandular expression and activity are highly correlated with the degree and severity of salivary gland damage and functional changes [9].

Prior studies have demonstrated that IFN-γ and IFN-α signaling, STAT1 and STAT4 protein activation, and increased Interleukin-33, MMP, and plasmin proteins all play a critical role in SjD pathology [22]. IFN-γ is secreted by CD4^+^ Th1 cells, natural killer (NK) cells, and CD8^+^ cytotoxic T cells, suggesting that ECM integrity disorders that often lead to SjD may start following lymphocytic responses to viral stimuli. Interestingly, STAT1 and STAT4 genes in SjD patients show abnormally high SNP concentrations compared to control subjects [23], which may influence higher levels of IL33 released in the extracellular space from damaged salivary cells upon pro-inflammatory stimulation of the epithelial barrier [22,24].

Previous studies by our team have shown MMP9 protein expression is upregulated by transcription factor ETS proto-oncogene 1 (ETS1) in immortalized SGECs (iSGECs) derived from female patients with salivary hypofunction [25] and is inhibited by activated STAT1/IRF1 signaling in human peripheral blood monocytes [26]. We also showed that ETS1 mRNA levels were increased in the salivary glands of female *sicca* and anti-SSA-positive and -negative SjD patients [25,27]. In another study, we demonstrated that ETS1 protein levels were also elevated in the labial salivary gland (LSG) tissue of *sicca* and anti-SSA-negative SjD patients within areas distant from immune cell infiltrates [28]. The mechanisms by which ETS1 is regulated in *sicca* and SjD patient LSGs are unknown. However, micro-RNAs (miRNAs) have been shown to regulate ETS1 protein expression with and without affecting mRNA abundance [29]. Specifically, miR-125b and miR-204 were shown to inhibit tumor growth in vivo by directly repressing ETS1 protein expression without altering mRNA abundance [30]. Conversely, mir-193b showed a negative correlation with ETS1 mRNA in hepatocellular carcinoma (HCC) suggesting that miR-193b mediates ETS1 mRNA degradation [31]. Furthermore, miR-145 was shown to directly target FLI1, a member of the ETS transcription factor family, by blocking migration in response to growth factors in colon cancer and pericytes [32]. The abundance and function of these miRNAs have not been studied in SjD or *sicca* patients.

The overall objective of this study was to validate the iSGECs derived from female sicca and SjD patients with salivary hypofunction as a Sjögren’s disease model through a targeted approach and explore mechanisms of SjD pathology through an unsupervised approach. Our aims were to (1) use a targeted approach to determine the effects on ETS1, STAT1, and IL33 mRNA and protein expression in iSGECs dosed with immunostimulators IFN-γ and toll-like receptor 3 agonist poly(I:C); (2) determine the effects of the immunostimulators on global mRNA expression and associated SjD pathways; and (3) examine miRNA regulators for genes that showed a protein level change in the absence of significant mRNA abundance change. The results from this study will further our understanding of Sjögren’s disease pathology and possibly offer valuable insight into classification markers and prevention strategies.

## 2. Results

**Figure 1:**
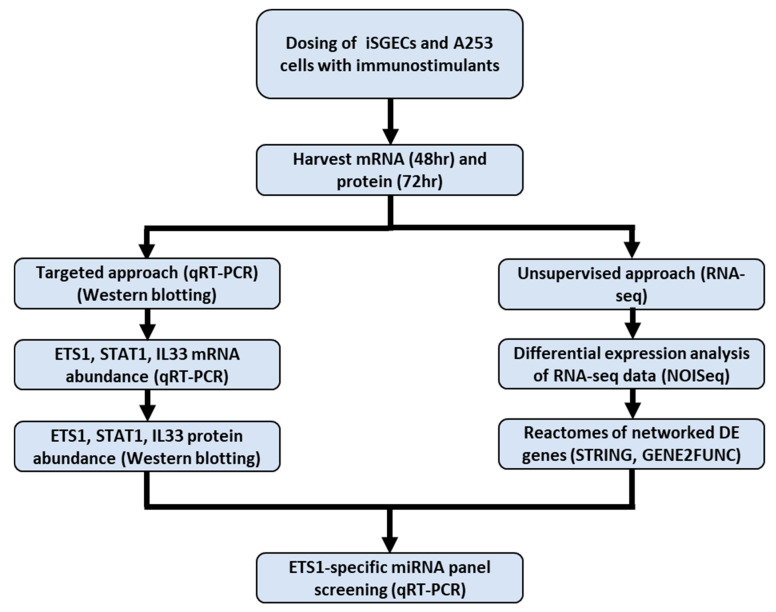
Overall experimental design flow chart. iSGECs were incubated for 48 and 72 h post-dosing. To assess the effects of immunostimulation, we followed a targeted approach to measure mRNA and protein abundance of candidate biomarkers. We also performed an unsupervised approach to assess transcriptome-wide effects between the two cell lines. miRNA screening for ETS1-specific miRNAs followed to assess their role in protein expression.

A flowchart showing the overall experimental design is presented in Figure 1.

### 2.1. Targeted Approach: SjD Pathogenesis Markers ETS1, STAT1, and IL33 Abundance in Immunostimulated iSGECs

**Figure 2:**
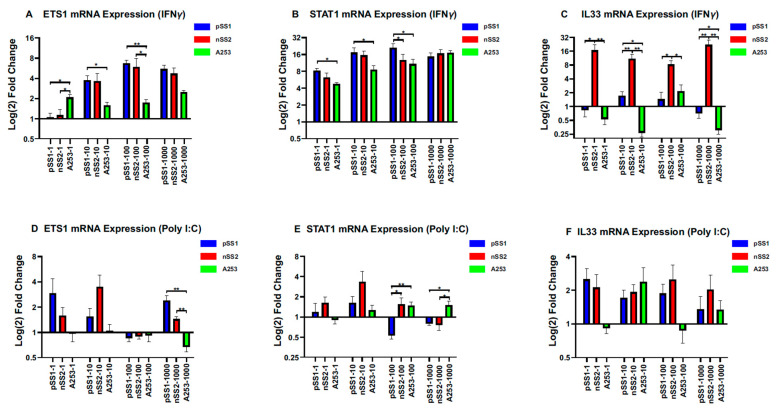
mRNA abundance of three candidate SjD biomarkers. qRT-PCR expressional analysis comparison of three genes previously shown to contribute to Sjögren’s syndrome progression. Bar graphs represent fold-change relative to the untreated control for (**A**) ETS1, (**B**) STAT1, and (**C**) IL-33 of IFN-γ-treated pSS1, nSS2, and A253 cell lines, and for (**D**) ETS1, (**E**) STAT1, and (**F**) IL-33 of poly(I:C)-treated PSS1, NSS2, and A253 cell lines. Expression levels were determined relative to GAPDH based on the ∆∆CT method using SYBR Green mix. Error bars represent means (±) standard error (SE) based on nine independent experimental replicates. Shapiro–Wilk’s test was used to determine data distribution. Mann–Whitney U-test (Bonferroni-corrected) was used to determine significance (** *p* < 0.01; * *p* < 0.05).

We closely examined the expression differences in ETS1, STAT1, and IL33 in both pSS1 and nSS2 cells by comparing them to control cell line A253 using qRT-PCR. ETS1 mRNA was significantly overexpressed (*p* < 0.05) at 10 ng/µL and above IFN-γ-treated nSS2 and pSS1 cells compared to A253. However, ETS1 was not differentially expressed in nSS2 vs. pSS1 cells (Figure 2A). pSS1 consistently showed higher STAT1 expression over A253 across low-mid-level IFN-γ treatments (Figure 2B). NSS2 cells showed dramatically higher IL33 expression, up to 16-fold greater than its respective control (Figure 2C). Poly(I:C)-treated cells (Figure 2D,F) showed increased levels of ETS1 and STAT1 with high dosing ranges in pSS1 and nSS2 cells compared to A253. However, IL-33 transcripts showed no significant upregulation in poly(I:C)-treated cell lines (Figure 2F). Cohen’s *d* and effect size correlation *r* reflects the significant results obtained (Supplementary Table S1).

### 2.2. ETS1, STAT1, IL33 Relative Protein Abundance in Immunostimulated iSGECs

**Figure 3:**
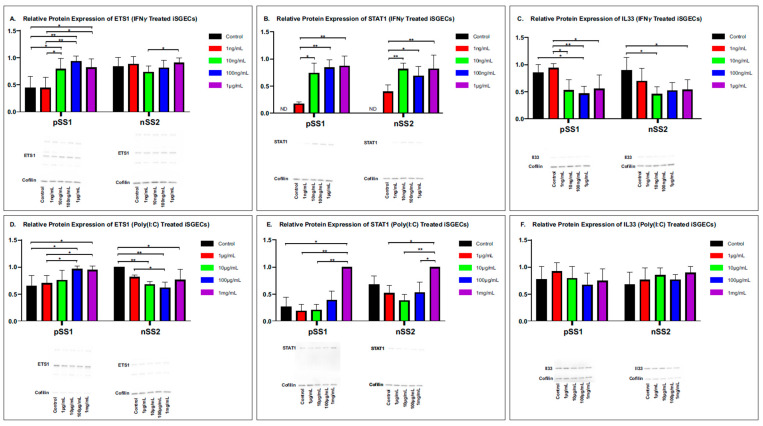
Protein abundance of three candidate SjD biomarkers. Active protein expression analysis of salivary gland cell lines treated with (**A**–**C**) IFN-γ and (**D**–**F**) Poly(I:C). Representative Western blots and semi-quantitative Western blot analysis (from 5 independent experimental replicates) are shown. Protein levels were determined 72 h post-dosing with IFN-γ and Poly(I:C) in pSS1 and nSS2 whole-cell lysates. Equal protein amounts were loaded in each lane and target bands were normalized to protein expression of cofilin. Error bars represent the mean (±) standard deviation. Loading controls and target proteins of the same blot were individually optimized for exposure requirements and reconstituted for imaging. Mann–Whitney U-test was used to determine significance between treatments (** *p* < 0.01; * *p* < 0.05). ND is not detected.

Following the mRNA expression determination of treated nSS2 and pSS1 cell lines, we used semi-quantitative Western blotting to examine the relative protein abundance between treatment groups (Figure 3). ETS1 protein abundance was significantly lower (*p* < 0.05) in the control and 1 ng/mL treatment groups, while the nSS2 cells only showed marginal significance (*p* = 0.042) for 10 ng/mL vs. 1 µg/mL (Figure 3A). Interestingly, the ETS1 protein levels in IFN-γ-treated pSS1 cells strongly correlate (Pearson’s correlation test, *p* < 0.01) to mRNA values obtained by qRT-PCR and RNA-seq, but no correlation is observed with nSS2 cell lines. STAT1 showed similar responses in both cell lines and protein levels strongly correlated with qRT-PCR and RNA-seq mRNA levels (Figure 3B). IL33 in pSS1 showed greater sensitivity to IFN-γ treatment specifically with low IFN-γ treatment, but both failed to significantly correlate with mRNA levels (Figure 3C).

Poly(I:C) treatment showed opposite trends between pSS1 and nSS2 cell lines, with protein expression increasing overall for pSS1 cells and decreasing for nSS2 cells compared to the control. Surprisingly, pSS1 protein levels showed strong correlation to RNA-seq mRNA readings but not qRT-PCR (*p* < 0.01; Figure 3D). Similar U-shaped responses were observed for STAT1, though nSS2 cells showed higher sensitivity at the low-mid dosing ranges. Both pSS1 and nSS2 protein levels determined by a Western blot analysis marginally correlated to RNA-seq and qRT-PCR values (*p* = 0.05 and *p* = 0.046, respectively; Figure 3E). IL33 failed to show significant protein expression changes when exposed to Poly(I:C) and did not correlate to mRNA values (Figure 3F).

### 2.3. Unsupervised Approach: RNA-Seq of Immunostimulated iSGECs

**Figure 4:**
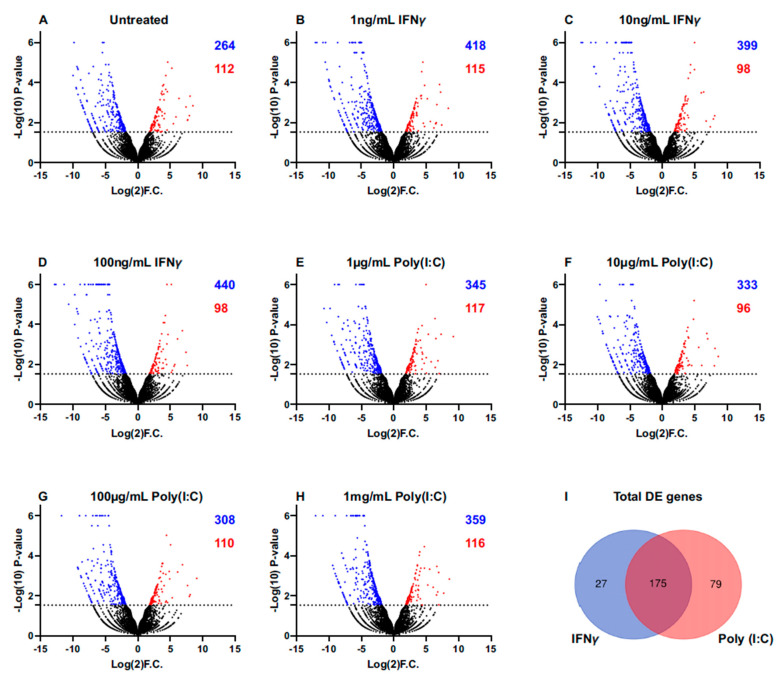
NOISeq differential expression analysis of immunostimulated nSS2 vs. pSS1 cell lines. (**A**–**H**) Volcano plots based on the NOISeq DE analyses comparing differentially expressed genes between nSS2 and pSS1 cell lines in the presence of (**A**) sham; (**B**) 1 ng/mL IFN-γ; (**C**) 10 ng/mL IFN-γ; (**D**) 100 ng/mL IFN-γ; (**E**) 1 µg/mL Poly(I:C); (**F**) 10 µg/mL Poly(I:C); (**G**) 100 µg/mL Poly(I:C); and (**H**) 1 mg/mL Poly(I:C) (*p* < 0.03). Blue dots represent single genes that are downregulated in comparison, while red dots represent upregulated genes by comparison. pSS1 cells showed about a 4-fold increase with IFN-γ treatments and about a 3-fold increase with Poly(I:C) treatments in upregulated DE genes compared to nSS2 cells. (**I**) Venn diagram showing conserved DE genes between the control and IFN-γ treatments (blue), and the control and Poly(I:C) treatments (red). In total, there were 175 conserved DE genes between all treatments and control.

We determined global gene expression profile comparisons of *sicca*- and SjD-derived iSGECs. The data was normalized for each sample’s expression profile to TMM to ensure gene expressions were directly comparable between and within samples. Overall, 376 genes were shown to be differentially expressed (DE) between untreated nSS2 and pSS1 cells at the 97% probability cut-off, with a majority (264) showing higher expression in pSS1 (blue dots, Figure 4A). Interestingly, pSS1 showed approximately 4-fold more DE genes expressed than nSS2 when treated with IFN-γ (Figure 4B,D), and 3-fold when treated with poly(I:C) (Figure 4E,H). In total, we found 175 DE genes between all IFN-γ and poly(I:C) treatments and the control (Figure 4I).

### 2.4. Reactome Identification of Conserved DE Genes

**Figure 5:**
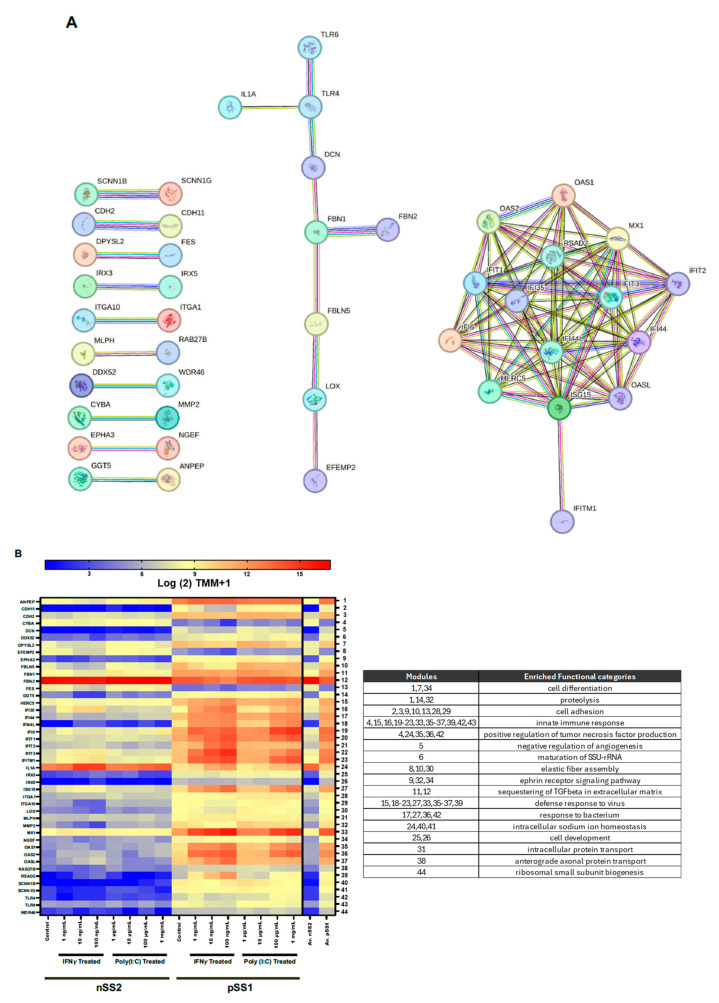
Network and mRNA abundance of conserved DE genes. (**A**) STRING network of DE genes conserved amongst all treatment groups and the control. Disconnected nodes were removed from the analysis. (**B**) Heat map showing expression of DE genes conserved amongst all treatment groups and the control. Genes are arranged in alphabetical order by their gene symbol and categorized by enriched gene ontology. Overall, most genes were shown to be upregulated in pSS1 cells except for CYBA, EFEMP2, FBN2, FES, and IL1A, involved in innate immunity, ECM integrity, cell adhesion, and homeostasis. Genes that demonstrated higher levels of interaction (i.e., more than one interaction) were all upregulated in pSS1 cells.

We further examined DE genes conserved across all treatments and the control at 97% confidence to examine gene interactions involved with SjD pathology. Of the 175 conserved DE genes, we found 44 interactors (Figure 5A). We also annotated the expressional patterns of gene interactors (Figure 5B) and significant reactome gene sets (*p*_adj_ < 0.05) using the gene2func database (Table 1). Our analysis showed all but five genes, IL1A, FES, FBN2, EFEMP2, and CYBA, were more abundant in nSS2 cells across all treatments. Two of these, FBN2 and EFEMP2, are extracellular matrix organization proteins involved with elastic fiber formation. Many genes involved with interferon signaling and antiviral immune responses, such as IFI44, IFIT proteins, MX1, OAS1, OAS2, OASL, and HERC5, were more abundant in pSS1 cells compared to nSS2 cells across all treatments and the control. Interestingly, MMP2, DCN, FBN1, and FBLN5, all involved in the breakdown of the extracellular matrix and interactors of STAT1, showed relatively low levels of expression in all nSS2 treatments.

### 2.5. Screening of a Panel of miRNAs to Determine the Effects on ETS1 Expression in iSGECs Dosed with Immunostimulators

**Figure 6:**
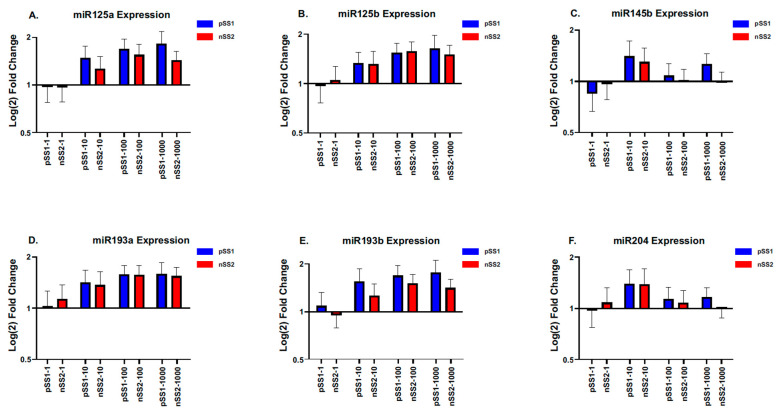
Abundance of ETS1-inhibiting miRNAs. Bar graphs represent fold-change relative to the control for (**A**–**C**) IFNγ-treated and (**D**–**F**) Poly(I:C)-treated pSS1 and nSS2 cell lines. Expression levels were determined relative to U6 based on the ∆∆CT method using SYBR Green mix. Error bars represent mean (±) standard error (SE) based on 15 independent experimental replicates. Mann–Whitney U-test was used to determine significance between treatment groups and a Wilcoxon signed-rank test was used to determine significance between pSS1 and nSS2 cell lines. Of interest, miR193b showed consistently higher expression in pSS1 cells and was highly significant (*p* < 0.01).

Following our ETS1 protein abundance correlation results, we measured the abundance of six miRNAs shown to regulate ETS1 mRNA expression without altering mRNA levels (Figure 6). Overall, we found no differential expression between pSS1 and nSS2 treatment groups. However, we found miR-193b was consistently expressed at lower levels in nSS2 cells (*p* < 0.01; Figure 6E). miR-145b also showed marginal significance between cell lines (*p* = 0.042; Figure 6C).

## 3. Discussion

This study is the first to utilize clinically relevant iSGEC cell lines derived from *sicca* and SjD patients to simultaneously determine the mRNA expression of pathogenesis specific genes as well as the global expression, in response to relevant immunostimulators. SjD pathology was previously shown to involve IFN-γ and IFN-α signaling, the activation of STAT proteins, the production of Interleukin-33, and the upregulation of MMPs and plasmin [22]. Although STAT1 showed no difference in mRNA or protein abundance between our cell lines, it is possible that a high number of SNPs could alter the function of STAT1 rather than its expression [23]. IL33 showed significantly higher mRNA expression in IFN-γ-treated nSS2 cells, while it expressed a significantly lower amount of protein compared to the control. Furthermore, the relative protein abundance of IL33 was significantly correlated between IFN-γ and poly(I:C) treatments, demonstrating a conserved response. The exact reason for this expression pattern remains unknown, though it is possible that pSS1 cells express higher levels of siRNA(s) targeting IL33 in the presence of IFN-γ [33]. For example, miR-29a was previously shown to regulate IL33 in tendon tissue remodeling [34] and to be expressed at higher levels in the saliva and salivary gland tissue of SjD patients compared to *sicca* patients [35]. Studies comparing the miRNA profiles of *sicca*- and SjD-derived iSGECs derived from a broad, yet well-defined spectrum of anti-SSA-negative and -positive patient cohort, would provide further insights on how to target SjD pathogenesis pathways. Such pathways may be targeted early on in the context of asynchrony of salivary gland loss of integrity over time, since patients developing SjD may have autoantibodies over 20 years before developing the disease [36].

Although pSS1 and nSS2 cells express similar levels of ETS1 mRNA in IFN-γ-treated iSGECs, a Western blot analysis demonstrated a different protein expression in pSS1 cells. Our miRNA analyses show that miR-145 and miR-193b, previously reported to regulate ETS1 through mRNA degradation [29], are expressed at higher levels in pSS1 cells. Despite such a result, mRNA and relative protein abundances were significantly correlated in pSS1 cells and not nSS2 cells, suggesting that some loss of miR-145 and miR-193b function occurs in pSS1 cells in response to IFN-γ. Although the reasoning for this is unclear, it is possible that alternative splicing events affect the maturation of miRNAs. In *Arabidopsis thaliana*, it was shown that miR-400 expression induced by heat stress caused a 100 bp excision, resulting in greater accumulation of miR-400 primary transcripts but lower levels of mature miR-400 [37]. Our RNA-seq results show that pSS1 cells have increased sensitivity to IFN-γ; therefore, they may be more prone to stress-induced alternative splicing events that result in immature miRNA production. It is also possible that stress-induced alternative splice variants of ETS1 accounting for 10% of total ETS1 in lymphocytes could bypass miRNA detection [38]. This possibility may provide an explanation for the lack of ETS1 mRNA-protein abundance correlation observed in nSS2 cells. However, alternative splicing effects on miRNA maturation have not been studied in a human disease model and remains speculative until validated. Studies quantifying alternative splice variants of ETS1, miRNA inhibition experiments, and large-scale miRNA expression profiles between *sicca* and SjD could further our understanding of SjD pathology.

Our unsupervised mRNA profiling approach identified 44 DE genes across all IFN-γ and poly(I:C) treatments. A majority of these were of higher abundance in pSS1 cells. However, five genes, CYBA, EFEMP2, FBN2, FES, and IL1A, involved in innate immunity, ECM integrity, cell adhesion, and homeostasis, were expressed at higher levels in nSS2 cells. Prior research has shown these genes play a critical role in connective tissue maintenance and macrophage activation [39,40]. The downregulation of these genes in pSS cells, especially CYBA, EFEMP2, and FES, possibly reflects involvement in SjD development through loss of function. Previous studies have proposed using some upregulated SjD genes, such as IFIT and MX1 proteins, involved with innate immune responses, as biomarkers for early onset of SjD [41,42]. Our experiment demonstrates that these genes can be used to distinguish between nSS2 and pSS1 cell lines. However, experiments involving more patient samples are needed to validate their clinical relevance.

Interestingly, IFIT1, IFIT2, and IFITM1 were shown to interact with several other conserved proteins such as nucleotidyl-transferases and HERC5. HERC5 was shown to exhibit antiviral activity towards ssRNA viruses such as HIV-1 and dsDNA viruses such as HPV [43,44], of which infection has been shown to increase risk of SjD development regardless of age [45]. Although nucleotidyl-transferases have not been studied in relation to SjD, several members of the 2′-5′-oligoadenylate synthetase (OAS) protein family, including OAS1, OAS2, and OASL, were shown to have several relevant protein interactions in our data. OAS1 activates RNase L enzymes in response to interferons, which degrades both endogenous and viral RNA [46].

These results indicate that the dysregulation of these genes may affect cellular function and promote lymphocytic migration to the salivary gland by promoting an overactive inflammatory immune response. Further examination of the genes identified regarding their role in SjD pathogenesis using a broader range of patient-derived iSGECs is needed for validation.

## 4. Materials and Methods

### 4.1. Cell Cultures and Response Curves to Immunostimulators

Immortalized salivary gland epithelial cell lines (iSGECs) nSS2 and pSS1, derived from a primary LSG culture of a non-SjD “*sicca*” female patient with a focus score (FS) of 0.16 and a female SjD patient with FS = 1.8, respectively, had been previously generated in our laboratory and characterized for acinar, ductal, and salivary function markers [47]. nSS2 and pSS1 cells were grown in Epi-life Basal media with HKGS supplements (Gibco, Waltham, MA, USA) and incubated at 37 °C with 5% CO_2_. A253 cells (ATCC, Manassas, VA, USA), originating from submaxillary salivary gland epidermoid carcinoma, were cultured per ATCC’s recommended protocol. Briefly, A253 cells were grown in McCoy’s 5A medium (VWR) supplemented with 10% FBS. All cell lines were starved of supplements for 24 h prior to experimentation.

pSS1, nSS2, and A253 cell growth curves were generated over a 72 h period to ensure optimal RNA and protein harvesting periods during the logarithmic growth period. Cells were seeded in triplicate (25% density; 6-well plate) and fixated (100% methanol; 12, 24, 48, and 72 h post-seeding) prior to staining and imaging under the microscope (0.1% crystal violet in PBS; 3 fields of view). Cell density was analyzed using ImageJ cell counter plugin [48]. Cells were dosed with 0-1-10-100-1000 ng/mL IFN-γ and 0-1-10-100-1000 µg/mL poly(I:C) immunostimulants over a 72 h period as previously described for other mammalian cell lines [49,50,51,52]. Control cell lines receiving no immunostimulation were dosed with sterile water. The cytotoxicity of dosing agents was determined with ATP CellTiter-Glo luminescent assays (Promega, Madison, WI, USA; six replicates every 12 h). Based on the results of the growth curve (Supplementary Figure S1A), all three cell lines were shown to be in logarithmic growth phase between 24 and 48 h. Luciferase assays show that IFN-γ (Supplementary Figure S1B) and poly(I:C) (Supplementary Figure S1C) show slight to no cytotoxic effects across all treatments on both pSS1 and nSS2 cells. Although IFN-γ caused 40–60% growth hinderance in A253 cells for all concentrations compared to untreated cells, RNA and protein yields were still within the acceptable range for downstream applications.

### 4.2. qRT-PCR and Reference-Based RNA-Seq

Data for qRT-PCR and reference-based RNA-seq analyses were obtained 48 h post-dosing based on the growth curves. RNA extraction was performed using *quick*-RNA prep kit (Zymo Research, Irvine, CA, USA) according to the manufacturer’s protocol. Samples were analyzed by using a Nanodrop lite plus spectrophotometer (ThermoScientific, Waltham, MA, USA) to ensure total RNA concentrations were above 150 ng for library prep and reverse transcription. For samples with no significant amount of DNA or protein contaminants, 500 ng RNA was reverse transcribed from each sample using the SmartScribe reverse transcriptase kit (Takara, Kusats, Shiga, Japan) following the manufacturer’s protocol. Random hexamers (IDT) and dNTP mix (NEB) were used. mRNA expression levels of ETS1, STAT1, and IL33 were determined relative to GAPDH, while miRNAs were determined relative to U6 small nuclear RNA 1 (U6) based on the ∆∆CT method using SYBR Green mix (Qiagen, Hilden, Germany). qRT-PCR primers are listed in Supplementary File S1.

RNA libraries were constructed using Illumina rRNA depletion kits (San Diego, CA, USA) according to the manufacturer’s protocol and quality checked using a bioanalyzer to ensure adequate cDNA was synthesized prior to sequencing. Sample reads were obtained using Illumina HiSeq 2 × 150 bp configuration (San Diego, CA, USA) to obtain at least 30 million reads per sample. Sequence quality was checked using FASTQC v0.11.9 program and sequence reads (i.e., all FASTQ files) were trimmed using TrimGalore v0.27. Adaptor sequences ‘AGATCGGAAGAGCACACGTCTGAACTCCAGTCAC’ were removed from forward reads and ‘AGATCGGAAGAGCGTCGTGTAGGGAAAGAGTGT’ adaptor sequences were removed from reverse reads. Samples were aligned to Human RefSeq GRCh38.p13 and a STAR index was created using the human genome for alignment. The alignment was completed for all treated and untreated samples using the splice-aware aligner. FeatureCounts was used to count genes from the annotated files utilizing GRCh38.p13 genomic annotation file to count sequence features with a minimum mapping quality score of 10. Due to the nature of the immortalized cell lines (i.e., derived from a single patient), the R package ‘NOISeq v2.52.0’ [53] was used to determine differential expression of treated vs. untreated samples using the built in ‘Trimmed Mean of M Component’ (TMM) normalization method with five simulated replicates each at 20% similarity as in previous studies [54,55,56]. Due to poor reads, data from cells treated with 1 µg/mL IFN-γ were removed from the analyses. Probabilities greater than 97%, to further prevent false positives, were used to determine differentially expressed genes, indicating that differential expressions were likely due to a change incurred by an experimental condition and not due to chance alone. pSS1 and nSS2 DE genes with greater than 97% probability conserved amongst all treatments and controls were examined to identify unique genes that separate *sicca*- and SjD-derived iSGEC cell lines. Networks were determined using the gene/protein network visualization program search tool for recurring instances of neighboring genes (STRING) [57]. Interacting genes were annotated with the GeneCodis database according to biological function [58]. Reactomes of networked genes were annotated with the GENE2FUNC database [59]. All scripts and accompanying RNA-seq data can be accessed through our lab’s GitHub repository (www.github.com/mbeckm01/SS_pathogenesis).

### 4.3. Semi-Quantitative Western Blotting

Treated and untreated cells were grown (6-well plate; 72 h) post serum-starving before harvesting whole-cell lysates for nuclear protein using Mammalian Protein Extraction Reagent (MPER; ThermoFisher, Waltham, MA, USA) for Western blotting. Primary antibodies were used (Supplementary File S1) prior to the use of anti-mouse IgG-HRP secondary antibodies. Supersignal West Pico solutions were used for signal detection (ThermoFisher). An Amersham ImageQuant 800 UV gel/blot Imaging System (Azure Biosystems, Dublin, CA, USA) was used for imaging. The quantification of ETS1, total STAT1, and IL-33 was performed using ImageJ 1.54.a, and all protein band intensities were normalized to cofilin expression. Bands were made relative to the highest intensity to compare abundance in each treatment group.

### 4.4. Statistical Analysis

Data distribution was assessed using a Shapiro–Wilk test prior to assessing statistical significance. Significant changes between and amongst immunostimulated cell lines in targeted mRNA abundance of ETS1, STAT1, and IL33 were analyzed using a Mann–Whitney U-test (9 replicates) (alpha = 0.05). Significant changes in relative protein abundance were determined using a Kruskal–Wallis test (5 replicates) (alpha = 0.05). Significant reactome gene sets from RNA-seq analyses were identified using a hypergeometric test with Benjamini–Hochberg adjustment (alpha = 0.05). Significant differences in normalized miRNA abundance between immunostimulated cell lines were analyzed by a Wilcoxon signed-rank test (15 replicates) (α = 0.05).

## 5. Conclusions

This study lays an accurate foundation for better understanding of SjD pathogenesis by highlighting SjD-relevant mRNA and protein abundance in response to immunostimulants, unique reactomes, and the differential miRNA abundance between *sicca*- and SjD-derived iSGECs. Because *sicca* patients are at an increased risk of developing SjD, iSGECs can provide highly relevant insight to SjD pathology. Future studies developing more representative SjD- and *sicca*-derived iSGECS and the validation of miRNA deregulations may provide further insights into SjD development.

## Figures and Tables

**Table 1:** Significant reactome gene sets of 44 interactive differentially expressed genes derived from the FUMA GENE2FUNC online tool. Significant reactome gene sets (*p*_adj_ < 0.05) identified comparing RNAseq data of pSS1 and nSS2 cell lines. ^a^ Significant pathways from our dataset that belong to the gene set. ^b^ Hypergeometric test with Benjamini–Hochberg adjustment *p*-value for genes within the gene set. ^c^ Entrez gene symbols identified from our dataset.

Gene Set ^a^	N	n	*p*_adj_-Value ^b^	Genes ^c^
INTERFERON ALPHA BETA SIGNALING	72	12	5.64 × 10^−18^	*ISG15*, *IFI6*, *RSAD2*, *IFIT2*, *IFIT3*, *IFIT1*, *IFITM1*, *OAS1*, *OAS2*, *OASL*, *IFI35*, *MX1*
INTERFERON SIGNALING	193	13	1.15 × 10^−14^	*ISG15*, *IFI6*, *RSAD2*, *HERC5*, *IFIT2*, *IFIT3*, *IFIT1*, *IFITM1*, *OAS1*, *OAS2*, *OASL*, *IFI35*, *MX1*
CYTOKINE SIGNALING IN IMMUNE SYSTEM	693	15	3.40 × 10^−1^	*ISG15*, *IFI6*, *RSAD2*, *IL1A*, *HERC5*, *IFIT2*, *IFIT3*, *IFIT1*, *IFITM1*, *OAS1*, *OAS2*, *OASL*, *MMP2*, *IFI35*, *MX1*
ANTIVIRAL MECHANISM BY IFN STIMULATED GENES	79	7	4.20 × 10^−8^	*ISG15*, *HERC5*, *IFIT1*, *OAS1*, *OAS2*, *OASL*, *MX1*
EXTRACELLULAR MATRIX ORGANIZATION	292	9	7.49 × 10^−7^	*ITGA10*, *ITGA1*, *LOX*, *FBN2*, *EFEMP2*, *DCN*, *FBLN5*, *FBN1*, *MMP2*
ELASTIC FIBER FORMATION	42	5	3.43 × 10^−6^	*LOX*, *FBN2*, *EFEMP2*, *FBLN5*, *FBN1*
OAS ANTIVIRAL RESPONSE	9	3	9.71 × 10^−5^	*OAS1*, *OAS2*, *OASL*
MOLECULES ASSOCIATED WITH ELASTIC FIBRES	35	4	9.71 × 10^−5^	*FBN2*, *EFEMP2*, *FBLN5*, *FBN1*
SENSORY PERCEPTION OF SALTY TASTE	6	2	8.28 × 10^−3^	*SCNN1G*, *SCNN1B*
NERVOUS SYSTEM DEVELOPMENT	543	7	8.28 × 10^−3^	*ITGA10*, *NGEF*, *EPHA3*, *ITGA1*, *DPYSL2*, *FES*, *MMP2*
CHL1 INTERACTIONS	8	2	1.34 × 10^−2^	*ITGA10*, *ITGA1*
DEGRADATION OF THE EXTRACELLULAR MATRIX	139	4	1.61 × 10^−2^	*FBN2*, *DCN*, *FBN1*, *MMP2*
SEMAPHORIN INTERACTIONS	64	3	2.72 × 10^−2^	*ITGA1*, *DPYSL2*, *FES*
PLATELET ADHESION TO EXPOSED COLLAGEN	15	2	3.91 × 10^−2^	*ITGA10*, *ITGA1*
CRMPS IN SEMA3A SIGNALING	16	2	4.17 × 10^−2^	*DPYSL2*, *FES*
INTEGRIN CELL SURFACE INTERACTIONS	82	3	4.59 × 10^−2^	*ITGA10*, *ITGA1*, *FBN1*
IRAK4 DEFICIENCY TLR2 4	18	2	4.68 × 10^−2^	*TLR6*, *TLR4*
INTERFERON GAMMA SIGNALING	86	3	4.69 × 10^−2^	*OAS1*, *OAS2*, *OASL*
REGULATION OF TLR BY ENDOGENOUS LIGAND	20	2	4.98 × 10^−2^	*TLR6*, *TLR4*
EPH EPHRIN SIGNALING	91	3	4.98 × 10^−2^	*NGEF*, *EPHA3*, *MMP2*

## Data Availability

All data used can be retrieved from the supplementary files or through our lab’s GitHub repository (www.github.com/mbeckm01/SS_pathogenesis).

## Supplementary Materials

The following supporting information can be downloaded at: https://www.mdpi.com/article/10.3390/ijms26125881/s1.
