# Supplementary material for: Elucidating Regulatory Mechanisms of Genes Involved in Pathobiology of Sjögren’s Disease: Immunostimulation Using a Cell Culture Model
Source: Int J Mol Sci. 2025 Jun 19;26(12):5881. doi: 10.3390/ijms26125881 (PMC12192667; doi:10.3390/ijms26125881)
Supplement: Supplementary file 1 [file ijms-26-05881-s001.zip › Supplementary Figure S1.pdf]

Supplementary Figure S1: iSGEC Growth and Immunostimulant Survivability

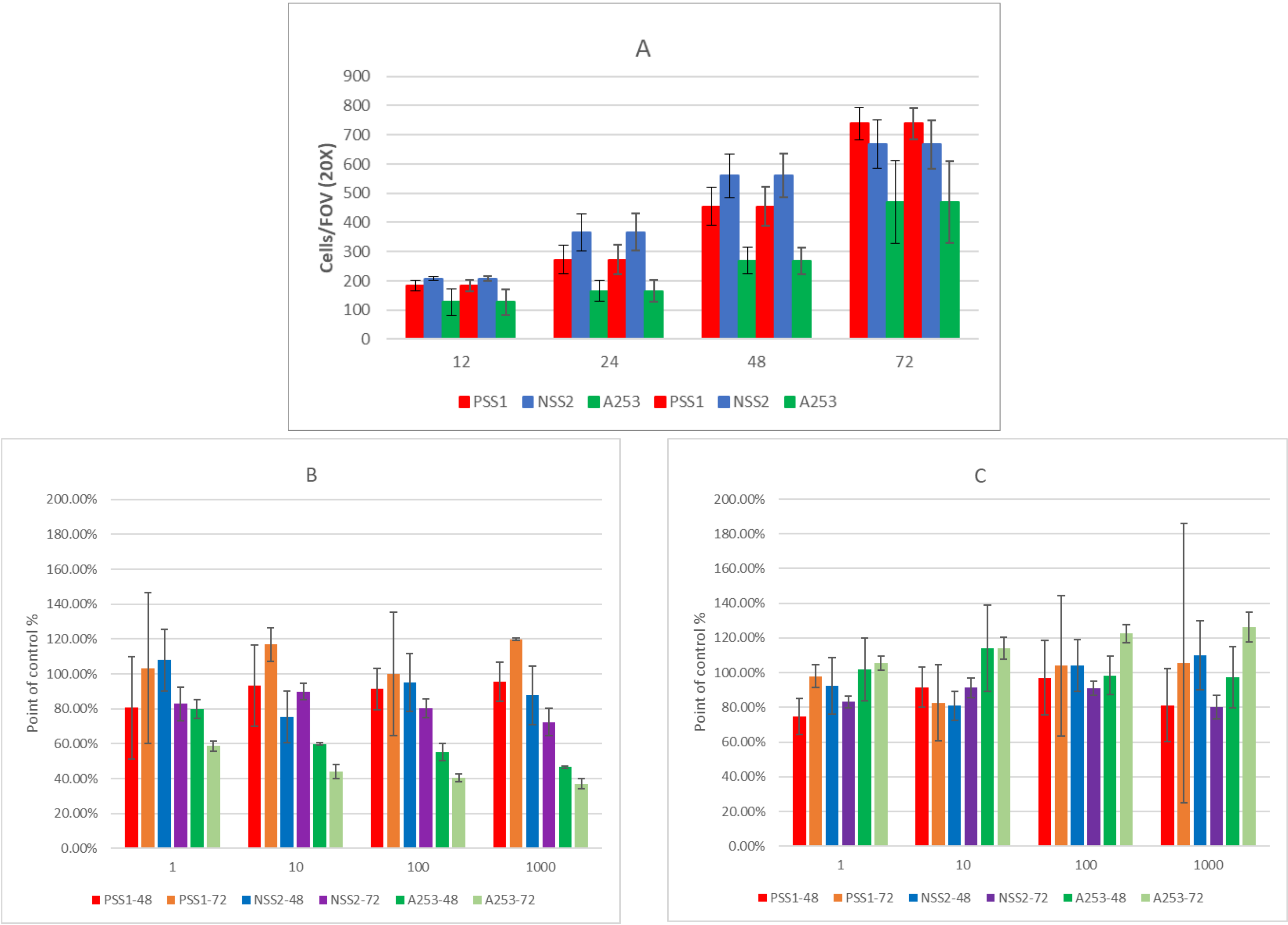

Supplementary Figure S1: iSGEC (pSS1 and nSS2) growth and immunostimulant survivability. (A) Cell growth rate following 72hrs growth. Growth curves were determined by ImageJ counts of 0.1x Crystal Violet stained plates. Curves represent the average +/- standard deviation of nine fields of view. (B-C) Luciferase assay of cell viability following 1, 10, 100, and 1000 ng/μL of (B) IFNγ and (C) Poly(I:C). Bars represent RLU luminescent average of six replicates +/- standard deviation.
